# P-1073. Developing Vaccines and Antibodies Targeting Conserved Peptidoglycan, Lipoteichoic Acid, and Lipopolysaccharide Epitopes to Combat Antimicrobial Resistance

**DOI:** 10.1093/ofid/ofae631.1261

**Published:** 2025-01-29

**Authors:** Clara J Sei, Nimisha Rikhi, Kellie Kroscher, Richard F Schuman, Kevin Muema, Aba Assiaw-Dufu, Gerald W Fischer

**Affiliations:** Longhorn Vaccines & Diagnostics, LLC, Gaithersburg, Maryland; Longhorn Vaccines & Diagnostics, LLC, Gaithersburg, Maryland; Longhorn Vaccines & Diagnostics, LLC, Gaithersburg, Maryland; Antibody and Immunoassay Consultants, Rockville, Maryland; Longhorn Vaccines & Diagnostics, LLC, Gaithersburg, Maryland; Longhorn Vaccines & Diagnostics, LLC, Gaithersburg, Maryland; Longhorn Vaccines & Diagnostics, LLC, Gaithersburg, Maryland

## Abstract

**Background:**

Antimicrobial resistance (AMR) poses a significant global health threat, driving the urgent need for non-antibiotic therapies such as vaccines and monoclonal antibodies (mAbs). Targeting conserved epitopes on gram-positive and gram-negative bacteria, including mycobacteria will be important for developing strategies against AMR. Key epitopes, including Peptidoglycan (PGN), Lipoteichoic acid (LTA), and Lipopolysaccharide (LPS) are crucial in this context. In this study, we illustrate the efficacy of a mAb directed against ultrapure PGN from *Staphylococcus aureus* (SA), alongside the capabilities of polyclonal serum antibodies induced by a multi-epitope composite peptide vaccine comprised of PGN, LTA, and LPS epitopes.

**Methods:**

MAb MD11 was produced from a mouse immunized subcutaneously with ultrapure PGN from SA and the mAb specificity identified using ultrapure PGN and a synthetic PGN peptide in a competitive inhibition assay. Additionally, polyclonal serum antibodies were derived from ICR mice immunized subcutaneously on days 0, 21, and 35 with 20 µg of multi-epitope composite peptide vaccine LPS.PGN.LTA05, formulated with AddaVax™ adjuvant. IgG1 titers to the immunogens and whole bacteria: SA, *Staphylococcus epidermidis*, *Mycobacterium smegmatis*, *Escherichia coli*, *B. subtilis*, Grp. B Streptococcus and killed *Mycobacterium tuberculosis* (MTB) were analyzed by ELISA.

**Results:**

MAb MD11 recognized and opsonized various gram-positive bacteria, including mycobacteria. PGN peptide competitively inhibited binding of MD11 to Ultrapure PGN, indicating specificity of MD11 to PGN epitope. Robust and broad polyclonal serum IgG1 antibodies recognizing both gram-positive and gram-negative bacteria, including mycobacteria were generated by the multi-epitope composite peptide vaccine.

**Conclusion:**

Anti-PGN mAb MD11 was broadly reactive to gram-positive bacteria. Polyclonal serum antibodies showed broad reactivity across both gram-positive and gram-negative bacteria, including mycobacteria. Developing vaccines and antibodies that target conserved bacterial epitopes may provide important strategies for prevention and treatment of bacterial infections, particularly for immunocompromised individuals, and could offer a useful approach to combat AMR.

**Disclosures:**

**All Authors**: No reported disclosures

